# Limb Regeneration in Amphibians: Immunological Considerations

**DOI:** 10.1100/tsw.2006.323

**Published:** 2006-02-26

**Authors:** Anthony L. Mescher, Anton W. Neff

**Affiliations:** Indiana University School of Medicine-Bloomington, Indiana University Center for Regenerative Biology and Medicine, Bloomington, IN 47405, USA

**Keywords:** regeneration, limb, nerves, wound epithelium, fibroblast growth factor, FGF, transferrin, inflammation, immunity, immunotolerance, wound repair

## Abstract

We review key aspects of what is known about limb regeneration in urodele and anuran amphibians, with a focus on the early events of the process that lead to formation of the regeneration blastema. This includes the role of the nerves and wound epithelium, but also covers the inflammatory effects of the amputation trauma and their importance for regenerative growth. We propose that immunotolerance is important for limb regeneration and changes in its regulation may underlie the loss of regenerative capacity during anuran metamorphosis.

## INTRODUCTION

Regeneration of amputated amphibian limbs is perhaps the most dramatic example of reparative growth among vertebrates. In anuran amphibians (frogs and toads), regeneration is restricted to the developing larval limb, but limb regeneration occurs throughout life in many, if not most, urodele species (newts and salamanders). Unlike fin and tail regeneration, the process in limbs includes reproduction, not only of a complex musculature and vasculature, but also a skeleton of articulated endochondral bones with the original anterior/posterior patterning of the autopods (hands or feet). Regeneration occurs via several overlapping phases, including wound closure, dedifferentiation, cell proliferation and migration, growth, patterning, and differentiation[1]. Understanding this process involves sorting out how the events elicited by the trauma of amputation set the stage for and integrate with the morphogenetic events and growth that allow the replacement structures of the limb to form.

It is now well established that the genes and at least the general patterning mechanisms required to reproduce missing limb components from an initial distal accumulation of cells, the regeneration blastema, in the limb stump are similar to those that directed limb ontology in the amphibian larva[2]. Detailed analysis of embryonic limb formation in the chick and mouse has been immensely successful in elucidating the signaling centers and molecular basis of this process[3]. A major issue in limb regeneration research is now and has long been to understand the cellular events that lead up to the blastema. Thirty years ago, Tassava and Mescher[4] proposed a general framework by which the major influences that allow limb regeneration are integrated and since then, work has not only confirmed those proposed roles, but has also provided the molecular mechanisms.

## REQUIREMENTS FOR LIMB REGENERATION

Classic studies from the 1920s through the 1960s showed that mesenchymal blastema cells arise locally by tissue and cell dedifferentiation rather than from circulating or tissue-specific stem cells (reviewed in [2]). Remodeling of the extracellular matrix (ECM), loss of tissue-specific histological features, and renewed proliferative activity begin during the inflammatory response to the trauma of amputation and do not require the signals for growth and patterning that maintain the blastema subsequently. Dedifferentiation in each of the cell types in limbs and their contribution to the blastema has been discussed elsewhere[5,6].

One poorly investigated aspect of limb regeneration that is likely of great importance to the overall process is the role of lymphocytes and other leukocytes emigrating to the site of injury during the earliest phase of regeneration. The systemic changes elicited by amputation and the effect on circulating white blood cells are indicated by differential counts of the cells during limb regeneration. After unilateral forelimb amputation in adult newts, the numbers of circulating lymphocytes and monocytes drop by more than 50% and do not return to normal levels until regeneration nears completion[7]. The injury may mobilize lymphocytes and macrophages to other sites besides the distal limb stump, but the presence of these cells in the early blastema has been noted in most earlier histological studies[8,9]. Hay and Fischman[10] estimated the number of *newly produced* monocytes and granulocytes alone in the newt limb stump during dedifferentiation (5 days after amputation) at 5000. While macrophages and neutrophils are no doubt employed to remove debris, dead cells, and bacteria, their other activities and the importance of the lymphocytes remain largely unknown.

Loss of tissue-specific gene expression and renewed cell cycling after injury occur routinely in cells of skin, various connective tissues, and Schwann cells in all vertebrates. What may be unique to amphibians is dedifferentiation and cellularization of the multinucleated fibers of skeletal muscle. Brockes and Kumar[6] found that cell cycle re-entry in newt myotubes is regulated by thrombin, a serine protease generated proteolytically during inflammation from the prothrombin in serum by the enzyme complex known as tissue factor. Thrombin-induced events in the myofiber nuclei include down-regulation of *Myf5*[11] and up-regulation of *Msx1*[12].

For the patterning events that direct formation of the blastema into the new limb, mesenchymal cells derived from fibroblasts appear to be of primary importance[5]. Though morphologically similar in various organs, “fibroblasts” are known to represent a heterogeneous set of connective tissue cells expressing a wide variety of genes unique to specific tissue microenvironments[13]. Early histological investigations of limb regeneration suggested that the first cells forming a recognizable blastema in the distal limb stump are fibroblasts derived from connective tissues of skin, muscles, nerves, and blood vessels (reviewed by [8,9]). Subsequent work confirmed the quantitative importance of cells from connective tissue, particularly the dermis, in the early blastema and suggested that interactions among these cells according to their positions and tissues of origin guide the subsequent development of other limb tissues in the regenerating limb[14,15]. Gardiner and colleagues[16] suggested that besides giving rise directly to the various connective tissue components of the new limb, these cells form a “blueprint” or scaffold that guides the subsequent immigration and growth of cells for the muscles, nerve sheathes, and blood vessels.

Schmidt[9] compares the early blastema of “fibroblasts” with the granulation tissue formed by fibroblasts in the early phase of mammalian wound repair. However, he points out a significant difference between the cells in the two systems with regard to their production of collagens and other ECM components. While formation of bundles of heavy collagen fibers dominates cutaneous repair in mammals, presumably to increase the tensile strength of the wound, in the growing amphibian blastema, collagen accumulation is limited to thin amorphous fibrils and there is little aggregation of isolated fibrils into coarser fibers until the reformation of muscle and cartilage proximally. Collagen turnover and fiber formation is regulated by collagenases and a variety of matrix metalloproteinases (MMP). Activities of these enzymes during dedifferentiation and blastema growth in amphibian limbs have been well studied[17] and inhibition of this activity has recently been shown to cause stunted or completely blocked limb regeneration in adult newts[18].

Once the earliest events involving tissue dedifferentiation, ECM remodeling, and cell cycle re-entry have been initiated as part of the response to amputation, growth of the regeneration blastema depends on factors released from two other sources: the apical "wound epidermis" and nerves. In amphibians capable of regeneration, skin wounds are closed by very rapid migration of a sheet of epidermal cells (keratinocytes) from the edges of the wound, rather than by the slower contraction of complete skin as occurs with cutaneous wounds of other vertebrates. In amputated limbs of newts, salamanders, and young anuran larvae, the cut surface is closed within hours by this "wound epithelium" or apical epidermal cap (AEC) which, lacking both a well-defined basement membrane and an underlying dermis, is in direct contact with the damaged tissues undergoing inflammation and beginning to dedifferentiate. The AEC soon becomes stratified and thickens as mesenchymal cells begin to accumulate directly underneath.

Classic work suggesting the AEC's histolytic function and possible role in tissue dedifferentiation (reviewed in [19]) is supported by the recent demonstration that several MMPs are expressed in basal cells of this structure[17,18]. The proposal that blastema cell accumulation beneath the AEC reflects its paracrine release of mitogens[4,20] is consistent with the presence of fibroblasts growth factors (FGF) in the epithelial tissue[15,21]. Thus, the AEC resembles the apical ectodermal ridge of the embryonic chick limb in its basic mechanism of promoting limb growth. FGF expression in both systems has been well studied. Genes for several FGFs are transcribed in both the AEC and the mesenchyme of the growing blastema[21,22], with the released proteins stored bound to heparin sulfates throughout the ECM[23]. Using two different experimental approaches *in vivo*, exogenous FGF has been shown to maintain blastemal growth in the absence of the AEC[24,25]. It now appears very likely that the AEC is of key importance for regeneration, both in its proteolytic effect on the ECM of the subjacent mesenchyme and its sustained mitogenic influence on that tissue's constituent cells.

The trophic influence of nerves on blastema growth emanates from the regenerating axons, sustaining both proliferation and hyaluronan production by mesenchymal cells[5,26]. Singer[27] showed that this neurotrophic effect is pan-neuronal, is up-regulated during axon regeneration, involves axonally transported proteins, and is required only during the early period of blastema growth. Brockes[28] subsequently demonstrated a subpopulation of blastema cells, possibly derived from the Schwann sheath, with persistent dependence on nerves for growth. One or more FGFs, which were originally purified from neural tissue, were proposed as mitogens for the effect[29,30]. However this is now considered unlikely, given the widespread nature of FGF expression within the blastema itself[21], its storage in heparin-containing matrices of the blastema[23], and its failure to exert any effect on blastema growth when presented on implanted beads *in vivo* in the absence of nerves[25].

During the nerve-dependent phase of blastema growth, axons penetrate throughout the mesenchyme, but capillaries are essentially absent except near the skin[31,32]. This raises the possibility that the axons provide factors required for cell proliferation that are otherwise delivered via the blood supply. The iron transport protein of plasma, transferrin, whose receptor-mediated uptake is the major mechanism by which cells obtain the ferric ion required for deoxyribonucleotide synthesis and maintenance of respiratory chain activity, is abundant in peripheral nerves. This factor undergoes anterograde axonal transport, is released at the distal ends of axons, and is lost from blastema tissue after denervation[33]. Moreover, the effects on cell growth when transferrin is removed from defined media, i.e., failure to complete DNA replication, followed by apoptosis, are also shown by dedifferentiating cells in denervated limb stumps[4,34]. Transferrin substitutes fully for the neural effect on DNA synthesis in cultured blastemas[35], although an effect on blastema growth has not been demonstrated *in vivo*. Nevertheless, the sum of data currently available suggests that axonal release of transferrin in the absence of an adequate supply of this factor from the blood can explain the neural dependence of cell proliferation in the early blastema[36].

Although most studies on dedifferentiation and the roles of the AEC and nerves have used urodeles (specifically newts and axolotls), it is clear that the general mechanism of regeneration is similar in anuran *Xenopus laevis* tadpole limbs. Epidermal migration and thickening, as well as cellular dedifferentiation and accumulation, occur in amputated limbs in this anuran species like those of urodeles[37]. In *Xenopus* blastemas, the distal epithelium and mesenchyme have been shown, respectively, to express FGF-8[38] and FGF-10[39]. Cells of *Xenopus* forelimbs denervated at the time of amputation exhibit normal dedifferentiation, including the early expression of *Prx1* and *Tbx5,* as well as cell cycle re-entry and the onset of DNA replication, but also undergo apoptosis without dividing[40]. Expression of *Fgf8*, *Fgf10*, and *Msx1* that normally begins later in regeneration was not seen in denervated/amputated limbs, suggesting that cell growth maintained by an adequate nerve supply is required for the activation of FGF signaling that mediates the epithelial-mesenchymal interactions effecting *Msx1* expression and limb outgrowth[40].

Slack[41] has offered the opinion that "in the molecular era, it is crazy to work on an amphibian other than *Xenopus*" since the number of genes cloned for this species far exceeds that of other amphibians. Techniques for making transgenic animals as well as commercially produced gene microarrays are available at this time only for *Xenopus* among amphibians. However, regeneration is only complete in anurans while the limbs are at an early stage of development and is grossly pattern deficient or completely lost in fully patterned, differentiating limbs. With these advantages and shortcomings in mind, we have turned to *Xenopus* to understand the loss of regenerative capacity in vertebrates phylogenetically different from urodeles.

## Loss of Regenerative Capacity in Anurans

Most amphibians develop limbs postembryonically, after several days or weeks as free-swimming larvae. In anurans that undergo metamorphosis, the major phase of hindlimb development coincides with the period of tail regression and other changes that lead to metamorphic climax. Amputation of the developing hindlimb in *Xenopus* during its early stages (from the small, round, limb bud to the first appearance of digit primordia; Nieuwkoop and Faber[42] stages 50–53) usually results in almost perfect reorganization and regrowth of the bud, followed by regeneration of a complete limb[37]. Amputation at progressively later stages of development produces increasingly incomplete regenerates. By the time five digits of the hindlimb are fully formed (stages 57–60) and thereafter, amputation typically is followed only by outgrowth of a cartilaginous "spike" covered by skin, but usually devoid of muscle[37]. Other anuran species display a similar sequence of regenerative loss, but do so more thoroughly than *Xenopus* and often form no outgrowth at all after amputation of a fully developed limb. At any stage of limb development, distal levels generally retain greater regenerative ability than more proximal levels, with this capacity retained longest at the levels of the joints[43].

Tassava and Olsen suggested that failure to produce a functional AEC or wound epithelium may explain the loss of regenerative ability in developing anuran hindlimbs[44]. Limbs incapable of regenerating a limb with normal morphology heal with epidermis that is quickly underlaid with connective tissue and fail to express FGF-8 and FGF-10[38,39]. Fibrotic activity occurs with little dedifferentiation and little cell proliferation. In species like *Xenopus* with pattern-deficient regeneration, fibroblastic cells may accumulate as a "pseudoblastema" from which the cartilaginous spike develops. The relative lack of dedifferentiation and failure to establish functional signaling centers in such stumps is concomitant with production of excess collagen and connective tissue[2].

That a general loss of regenerative capacity occurred during the phylogenic development of vertebrates is well established[45]. What is less well understood by regeneration biologists is that decreasing regenerative potential correlates broadly with refinements of the organisms' adaptive immunity. Although all (jawed) vertebrates express the basic components of the adaptive immune system (T, B, and regulatory lymphocytes; the major histocompatibility complexes; one or more immunoglobulins), urodeles appear to be much less efficient in their use. Transplanted tissues in urodeles are rejected only very slowly and antibody production (IgM only) is slow and inefficient[46,47,48].

A wealth of immunological evidence indicates that adult anurans, which invariably regenerate limbs more poorly than urodeles[49], possess much more efficient adaptive immunity than urodeles[46].

Among adult anurans, *Xenopus* and *Bombina* species, which are members of phylogenetically more primitive families[50], regenerate amputated limbs more fully than less primitive *Rana* species[49]. Comparative immunologists indicate that several anatomical features of the immune system and characteristics of adaptive immunity are more advanced in *R. pipiens* than in *X. laevis*[51]. Thus, across the spectrum of amphibian species whose immune systems have been examined, there seems in general to be an inverse relationship between the sophistication of adaptive immunity and the capacity to regenerate limbs.

The premetamorphic and prometamorphic period in anurans during which regenerative capacity disappears is a time in which the adaptive immune system, along with nearly all other organ systems, is undergoing profound changes and becoming more efficient. Compared to larvae, postmetamorphic *Xenopus* reject skin allografts much more rapidly, produce antibodies of greater diversity and binding affinity, and have more effective cytotoxicity for tumor cells[52]. A major turnover of circulating lymphocytes occurs during metamorphosis, leading to self/non-self incompatibility between larval and adult tissues in the same animal. This is indicated both by mixed lymphocyte reactions[53] and by observations that *Xenopus* skin removed from larvae, cryopreserved, and then grafted back to the same donors after metamorphosis is rejected acutely[54,55].

Anurans likely use several strategies to avoid self-destruction by autoimmune mechanisms during the transition from larval to adult tissues, but the best studied is the dramatic rise in corticosteroid hormone levels that accompanies late metamorphosis[52]. Transiently high concentrations of corticosterone may induce apoptosis of larval lymphocytes[52] as well as inhibit the expansion and function of newly formed adult T cells[56]. During the metamorphic period, allogeneic skin grafts are tolerated much longer than similar grafts made during, before, or after metamorphosis[57]. While the adaptive immune system is closely regulated to prevent autoimmunity during the tissue changes that accompany the larval-to-adult transition, the ongoing need for protection against pathogens may be afforded primarily by the organism’s increasingly important innate immunity, including the supply of potent antimicrobial peptides characteristic of adult skin[57].

Although generalized immunosuppression by corticosteroids appears to be important during metamorphosis, autoimmunity against newly appearing, adult-type cells and tissues can also be inhibited locally. Ono and Tochinai[58] demonstrated that “semi-xenogeneic” skin grafts (from *X. laevis – X. borealis* hybrids) on stage 53–54 *X. laevis* actively induce local tolerance to the graft. If the hosts first undergo thymectomy, tolerance does not occur, as shown previously by Cohen’s and Du Pasquier’s groups. The tolerance-inducing activity is due to lymphoid cells in both the dermis and epidermis and about 30% of these cells stain with a marker for T cells. These cells can be isolated from the skin grafts and used to induce tolerance in a new host. Intraperitoneal injection of as few as 100 graft-derived lymphoid cells induces tolerance to similar grafts in new hosts at this stage[58].

Ono and Tochinai[58] suggest that lymphoid cells extracted from tolerated skin grafts represent another fail-safe mechanism against autoimmunity during metamorphosis by inducing local tolerance of adult-type antigens as they appear. The concept proposed by these authors is basically that of peripheral self-tolerance, which has been widely investigated in mice and men, and is now a well-established aspect of adaptive immunity[59]. We have suggested that greater understanding of peripheral self-tolerance as it occurs in immunologically more primitive vertebrates, such as amphibians, is likely to yield new insights not only into how autoimmunity is avoided during metamorphosis, but also into why new formation of adult-type tissues by regeneration is restricted to such vertebrates[60].

## PERIPHERAL SELF-TOLERANCE AND THE INJURY EFFECT

The concept of peripheral tolerance, which a new textbook refers to as “the final frontier of immunology”[59], has forced reconsideration of previous assumptions about how autoimmunity is normally avoided. It is now clear that potentially autoreactive T and B cells are not uniformly removed in the thymus and bone marrow (central immune organs) and are common in the circulation. What prevents such cells from becoming activated and attacking self-antigens? Moreover, why is there no immunological attack to those self-antigens acquired only long after birth or late in larval development, such as antigens of developing anuran limbs?

The response of a naïve autoreactive T cell when it meets its cognate antigen in the periphery — to be activated and attack self-tissues or to be “tolerized” and rendered harmless — is determined by a complex interaction with the antigen presenting cell (APC) and the cytokine microenvironment of the interacting cells, which includes factors released from those cells as well as cells that comprise the tissue itself[59]. The most common APCs on which naïve, potentially autoreactive T cells meet their antigens are “immature” dendritic cells (DCs): monocyte-derived cells that reside in many connective tissues and are specialized for antigen presentation to T cells. Immature DCs are those that do not express surface costimulatory molecules on specific binding to a T cell. Without these costimulators, the naïve T cell interacting with an immature DC undergoes tolerization, not activation. The current view is that immature DCs appear to be the prime inducers of peripheral tolerance.

However, in response to tissue injury or infection, the residential DCs mature and do express costimulatory factors on T cell binding. In injuries and certain similar situations for tissues, a variety of “danger signals” are released from damaged and necrotic cells or from invading microorganisms. The most important such signals include various “heat shock proteins” released from lysed cells and various cell wall components of bacteria. Danger signals bind receptors on innate immune system cells (including macrophages and neutrophils) to induce secretion of various inflammatory cytokines which, among other activities, trigger bacterial killing, debris removal, and the onset of wound-repair activities. The danger signals and new cytokines also induce expression of the costimulatory factors on DCs bound to T cells, after which tolerization does not occur and the T cells are activated to participate in fighting infections and repairing tissue damage. If potentially self-reactive lymphocytes are present, their activation can lead to autoimmunity and, in fact, many autoimmune disorders appear after subtle injuries or infections[59].

That tissue injuries do not usually produce autoimmunity reflects the fact that most lymphocytes specific for self-antigens are eliminated centrally, during their initial production in the thymus. With newly expressed antigens that appear only in organs developing well after the onset of lymphocyte formation in the thymus, such as the factors regulating formation of anuran limbs, the likelihood of self-reactive T and B cells and the potential for autoimmunity emerging in the context of tissue injury may be greater.

## IMMUNITY AND REGENERATION

The newly recognized importance of peripheral tolerance and other aspects of both innate and adaptive immunity offer new ways to approach questions about tissue dedifferentiation, the onset of limb regeneration, and the loss of such regenerative capacity in vertebrates with more sophisticated immune systems. Considerations of an immunological influence in limb regeneration are not new[7,61], but occurred before the local regulatory interactions of lymphocytes and APCs in the skin were understood and before the importance of DCs in producing local immunotolerance was appreciated. With the importance of peripheral immunity now well appreciated, the observations discussed by earlier investigators should be revisited.

Immune cell interactions may be of particular importance in amphibians since neither urodeles nor certain anurans (such as *Xenopus*) have lymph nodes[59]. Antigen presentation and tolerization in such vertebrates may occur entirely within the skin and other peripheral tissues, without the involvement of lymph node microenvironments as in mammals. As shown in Fig. 1, various APCs are present in amphibian skin, including Langerhans cells in the epidermis[62] and MHC class II positive DCs in tadpole skin[63]. We have found Langerhans cells in the epidermis and wound epithelia of regenerating limbs in both urodeles and *Xenopus* larvae (manuscript in preparation). Langerhans cells are primarily tolerogenic, preventing skin hypersensitivity[63], and the possibility of a role for these cells in setting the conditions for the blastema to form should be examined.

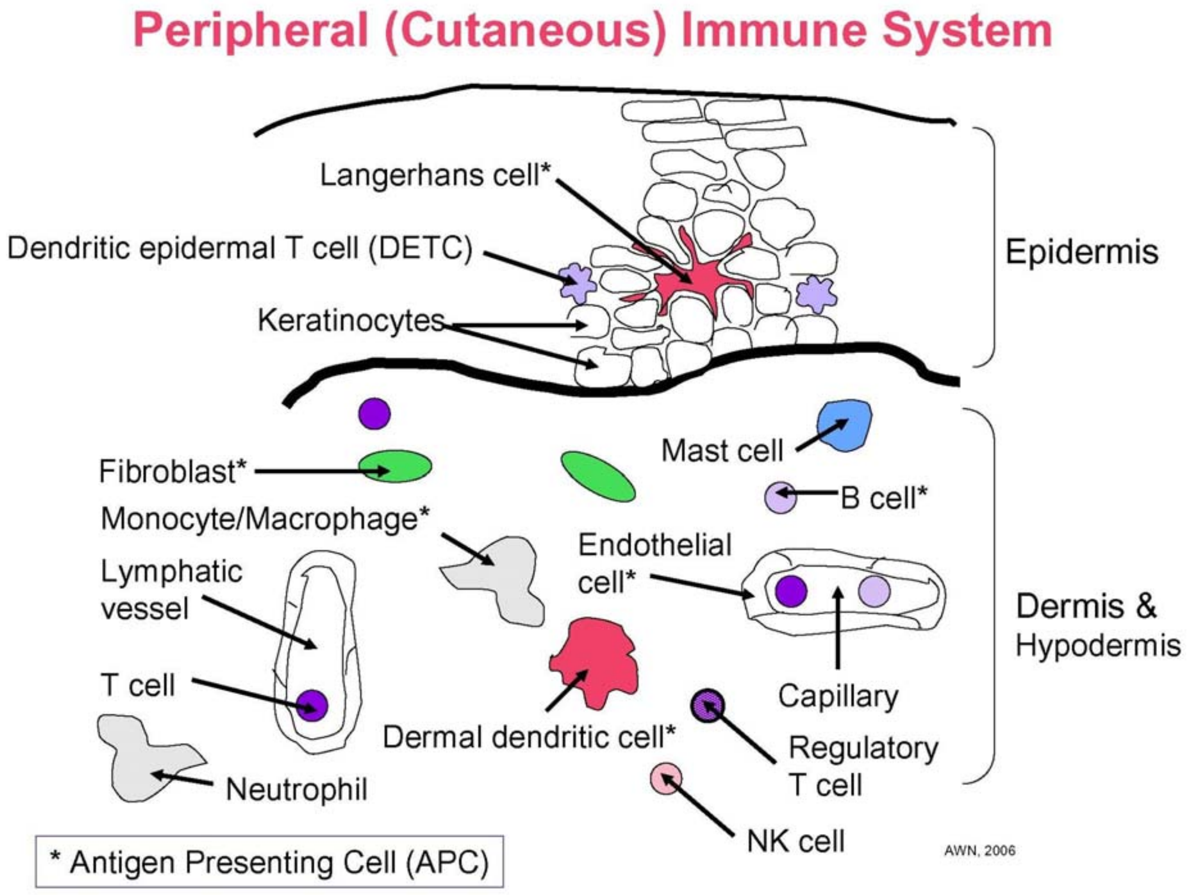

**FIGURE 1.** Cells of the peripheral immune system in amphibian skin. Although not well studied in urodeles, postmetamorphic anurans appear to have most components of cutaneous immunity found in mammalian skin. (Modified from [60].)

The activities of lymphocytes are also influenced by components of the innate immune system, including complement. Complement factors C3 and C5 are synthesized locally by cells of regenerating limbs[65,66], along with several other components of the complement system (manuscript in preparation). The membrane-bound regulator of complement activity, CD59, is also expressed in the blastema and has been implicated in local cell-cell interactions mediating positional identity[67]. Further work may reveal that complement components, as well as activators and inhibitors of complement function, have a variety of functions in the amphibian limb stump, including a tolerogenic function important for establishment of the blastema[68].

If it is important for regeneration, one might expect local immunotolerance or immune suppression to be demonstrably "stronger" in limbs of urodeles than in the nonregenerating limbs of anurans. Experimental immunoadjuvants, compounds that elicit a strong inflammatory response and generally produce fibrosis, would therefore be expected to be more effective in anurans than urodeles. One such adjuvant is beryllium[69], which has been shown to inhibit urodele limb regeneration completely when applied in solution to the limb stump[70]. With limb stumps of *Xenopus* larvae, local beryllium treatment inhibits regeneration at a tenfold lower dose than that required for a similar effect in urodeles and also produces an overt inflammatory response never seen with urodeles (manuscript in preparation). Such results are consistent with the hypothesis that peripheral immunity is regulated differently in the two amphibians, with greater immunotolerance in the limbs of urodeles.

The immune system may even affect the role of nerves in limb regeneration. As recently shown by several investigators[71,72] various immunological activities, especially functions of APCs and other cells involved in peripheral immunity, are modified by sensory, motor, and autonomic nerves. Thus, the possibility exists that the crucial growth-promoting influence produced by nerves in the early blastema may include neuropeptides exerting their effects via various cells of the immune system. One such neuropeptide, substance P, which has been implicated in blastema growth[2], exerts a wide variety of effects locally on immune cells and many other such peptides exist[71].

## CONCLUSION

In summary, limbs of urodeles and larval anurans appear to regenerate with the same general mechanism after amputation. Epidermal closure of the wound produces a dermis-free wound epithelium, beneath which tissues undergo dedifferentiation and cells accumulate to form the regeneration blastema. Cells of the blastema have positional identity relative to one another and their growth re-establishes the pattern of the missing limb. In adult anuran limbs that do not regenerate, wound closure by epidermis is concurrent with excessive fibrotic activity which appears to preclude functionality of a potential AEC/wound epithelium. Signaling centers are not established and a blastema capable of forming a normal limb does not arise.

The loss of regenerative ability in anuran limbs coincides with major changes in the immune system, including development of increasingly functional adaptive immunity. Urodeles, which generally regenerate limbs very well, display relatively inefficient adaptive immunity, with poor allograft rejection. Such observations, together with recent work on mammalian models of regeneration that have implicated immunity (reviewed [73]), have led to renewed interest in how cells of the immune system could be involved in determining the success or failure of limb regeneration[60]. Further investigation of peripheral immunity and immunotolerance in amphibians should provide new insights for understanding regenerative capacity in these organisms.

## ACKNOWLEDGMENTS

The authors wish to thank members of the Indiana University Center for Regenerative Biology and Medicine for helpful discussions and the Indiana 21st Century Fund for support of the current research in our labs cited here.

## References

1. Endo, T., Bryant, S.V., and Gardiner, D.M. (2004) A stepwise model system for limb regeneration. *Dev. Biol.* **270,** 135–145.
2. Stocum, D. (1995) *Wound Repair, Regeneration and Artificial Tissues*. R.G. Lander, Austin.
3. Tickle, C. (2003) Patterning systems - from one end of the limb to the other. *Dev. Cell.* **4**, 449–458.
4. Tassava, R.A. and Mescher, A.L. (1975) Roles of injury, nerves, and wound epidermis during initiation of amphibian limb regeneration. *Differentiation* **4**, 23–24.
5. Mescher, A.L. (1996) The cellular basis of limb regeneration in urodeles. *Int. J. Dev. Biol.* **40,** 785–795.
6. Brockes, J.R. and Kumar, A. (2002) Plasticity and reprogramming of differentiated cells in amphibian regeneration. *Nat. Rev. Mol. Cell Biol.* **3**, 566–574.
7. Sicard, R. (1985) Leukocytic and immunological influence on regeneration of amphibian forelimbs. In *Regulation of Vertebrate Limb Regeneration.* Sicard, R., Ed. Oxford University Press, New York. pp. 128–145.
8. Chalkley, D. (1959) The cellular basis of limb regeneration. In *Regeneration in Vertebrates.* Thornton, C., Ed. University of Chicago Press, Chicago. pp. 34–58.
9. Schmidt, A. (1968) *Cellular Biology of Vertebrate Regeneration and Repair*. University of Chicago Press, Chicago.
10. Hay, E. and Fischman, D. (1961) Origin of the blastema in regenerating limbs of the newt *Triturus viridescens*. *Dev.*

*Biol.* **3**, 26–59.

11. Imokawa, Y., Gates, P.B., Chang, Y.T., Simon, H.G., and Brockes, J.P. (2004) Distinctive expression Myf5 in relation to differentation and plasticity of newt muscle cells. *Int. J. Dev. Biol.* **48**, 285–291.
12. Kumar, A., Velloso, C.P., Imokawa, Y., and Brockes, J.P. (2004) The regenerative plasticity of isolated urodele myofibers and its dependence on Msx1. *PloS Biol.* **2**, 1168–1176.
13. Chang, H.Y., Chi, J.T., Dudoit, S., Bondre, C., van de Rijn, M., Botstein, D., and Brown, P.O. (2002) Diversity, topographic differentiation, and positional memory in human fibroblasts. *Proc. Natl. Acad. Sci. U. S. A.* **99,** 12877–12882.
14. Gardiner, D. and Bryant, S. (1989) Organization of positional information in the axolotl limb. *J. Exp. Zool.* **251**, 47–55.
15. Han, M., Yang, X., Taylor, G., Burdsal, C.A., Anderson, R.A., and Muneoka, K. (2005) Limb regeneration in higher vertebrates: developing a roadmap. *Anat. Rec.* **287B**, 14–24.
16. Gardiner, D.M., Endo, T., and Bryant, S.V. (2002) The molecular basis of amphibian limb regeneration: integrating the old with the new. *Semin. Cell Dev. Biol.* **13,** 345–352.
17. Yang, E.V., Gardiner, D.M., Carlson, M.R.J., Nugas, C.A., and Bryant, S.V. (1999) Expression of Mmp-9 and related matrix metalloproteinase genes during axolotl limb regeneration. *Dev. Dynam.* **216**, 2–9.
18. Vinarsky, V., Atkinson, D.L., Stevenson, T.J., Keating, M.T., and Odelberg, S.J. (2005) Normal newt limb regeneration requires matrix metalloproteinase function. *Dev. Biol.* **279,** 86–98.
19. Singer, M. and Salpeter, M. (1961) Regeneration in vertebrates: the role of the wound epithelium. In *Growth in Living Systems.* Zarrow, M., Ed. Basic Books, New York. pp. 277–311.
20. Mescher, A.L. (1976) Effects on adult newt limb regeneration of partial and complete skin flaps over amputation surface. *J. Exp. Zool.* **195**, 117–127.
21. Christensen, R.N., Weinstein, M., and Tassava, R.A. (2001) Fibroblast growth factors in regenerating limbs of *Ambystoma*: cloning and semi-quantitative RT-PCR expression studies. *J. Exp. Zool.* **290**, 529–540.
22. Giampaoli, S., Bucci, S., Ragghianti, M., Mancino, G., Zhang, F., and Ferretti, P. (2003) Expression of FGF2 in the limb blastema of two *Salamandridae* correlates with their regenerative capability. *Proc. R. Soc. Lond. Ser. B Biol. Sci.* **270**, 2197–2205.
23. Boilly, B., Cavanaugh, K.P., Thomas, D., Hondermarck, H., Bryant, S.V., and Bradshaw, R.A. (1991) Acidic fibroblast growth-factor is present in regenerating limb blastemas of axolotls and binds specifically to blastema tissues. *Dev. Biol.* **145**, 302–310.
24. Chew, K.E. and Cameron, J.A. (1983) Increase in mitotic-activity of regenerating axolotl limbs by growth factor-impregnated implants. *J. Exp. Zool.* **226**, 325–329.
25. Dungan, K.M., Wei, T.Y., Nace, J.D., Poulin, M.L., Chiu, I.M., Lang, J.C., and Tassava, R.A. (2002) Expression and biological effect of urodele fibroblast growth factor 1: relationship to limb regeneration. *J. Exp. Zool.* **292**, 540–554.
26. Mescher, A.L. and Cox, C.A. (1988) Hyaluronate accumulation and nerve-dependent growth during regeneration of larval ambystoma limbs. *Differentiation* **38**, 161–168.
27. Singer, M. (1978) Nature of the neurotrophic phenomenon in urodele limb regeneration. *Am. Zool.* **18,** 829–841.
28. Brockes, J.P. (1984) Mitogenic growth-factors and nerve dependence of limb regeneration. *Science* **225**, 1280–1287.
29. Gospodarowicz, D. and Mescher, A.L. (1980) Fibroblast growth factor and the control of vertebrate regeneration and repair. *Ann. N. Y. Acad. Sci.* **339**, 151–174.
30. Mullen, L.M., Bryant, S.V., Torok, M.A., Blumberg, B., and Gardiner, D.M. (1996) Nerve dependency of regeneration: the role of distal-less and FGF signaling in amphibian limb regeneration. *Development* **122**, 3487–3497.
31. Peadon, A.M. and Singer, M. (1966) Blood vessels of regenerating limb of adult newt, Triturus. *J. Morphol.* **118**, 79–89.
32. Rageh, M.A.E., Mendenhall, L., Moussad, E.E.A., Abbey, S.E., Mescher, A.L., and Tassava, R.A. (2002) Vasculature in pre-blastema and nerve-dependent blastema stages of regenerating forelimbs of the adult newt, *Notophthalmus viridescens*. *J. Exp. Zool.* **292**, 255–266.
33. Kiffmeyer, W.R., Tomusk, E.V., and Mescher, A.L. (1991) Axonal-transport and release of transferrin in nerves of regenerating amphibian limbs. *Dev. Biol.* **147**, 392–402.
34. Mescher, A.L., White, G.W. and Brokaw, J.J. (2000) Apoptosis in regenerating and denervated, nonregenerating urodele forelimbs. *Wound Repair Regen.* **8**, 110–116.
35. Mescher, A.L., Connell, E., Hsu, C., Patel, C., and Overton, B. (1997) Transferrin is necessary and sufficient for the neural effect on growth in amphibian limb regeneration blastemas. *Dev. Growth Differ.* **39**, 677–684.
36. Dinsmore, C. and Mescher, A. (1998) The role of the nervous system in regeneration. In *Cellular and Molecular Basis of Regeneration: From Invertebrates to Humans.* Ferretti, P. and Geraudie, J., Eds. John Wiley & Sons, Chichester. pp. 79–110.
37. Dent, J.N. (1962) Limb regeneration in larvae and metamorphosing individuals of South African clawed toad. *J. Morphol.* **110**, 61–78.
38. Christen, B. and Slack, J.M.W. (1997) FGF-8 is associated with anteroposterior patterning and limb regeneration in Xenopus. *Dev. Biol.* **192**, 455–466.

39. Yokoyama, H., Yonei-Tamura, S., Endo, T., Belmonte, J.C.I., Tamura, K., and Ide, H. (2000) Mesenchyme with fgf-10 expression is responsible for regenerative capacity in Xenopus limb buds. *Dev. Biol.* **219**, 18–29.
40. Suzuki, M., Satoh, A., Ide, H., and Tamura, K. (2005) Nerve-dependent and -independent events in blastema formation during *Xenopus* froglet limb regeneration. *Dev. Biol.* **286**, 361–375.
41. Slack, J. (2000) The *Xenopus* tadpole - a new organism for regeneration research. *e-biomed.* **1**.
42. Nieuwkoop, P. and Faber, J. (1967) *Normal Table of Xenopus laevis (Daudin)*. North-Holland, Amsterdam.
43. Wolfe, A. D., Nye, H.L.D., and Cameron, J.A. (2000) Extent of ossification at the amputation plane is correlated with the decline of blastema formation and regeneration in *Xenopus laevis* hindlimbs. *Dev. Dynam.* **218**, 681–697.
44. Tassava, R.A. and Olsen, C.L. (1982) Higher vertebrates do not regenerate digits and legs because the wound epidermis is not functional - a hypothesis. *Differentiation* **22,** 151–155.
45. Thouveny, Y. and Tassava, R. (1998) Regeneration through phylogenesis. In *Cellular and Molecular Basis of Regeneration: From Invertebrates to Humans.* Ferretti, P. and Geraudie, J., Eds. John Wiley & Sons, Chichester.. pp. 9-43.
46. Flajnik, M., Miller, K., and Du Pasquier, L. (2003) Evolution of the immune system. In *Fundamental Immunology.* Paul, W., Ed. Lippincott Williams & Wilkins, Philadelphia. pp. 510–570.
47. Tournefier, A., Laurens, V., Chapusot, C., Ducoroy, P., Padros, M.R., Salvadori, F., and Sammut, B. (1998) Structure of MHC class I and class II cDNAs and possible immunodeficiency linked to class II expression in the Mexican axolotl. *Immunol. Rev.* **166**, 259–277.
48. Cohen, N. (1971) Amphibian transplantation reactions: a review. *Am. Zool.* **11,** 193–205.
49. Scadding, S.R. (1977) Phylogenic distribution of limb regeneration capacity in adult *Amphibia*. *J. Exp. Zool.* **202**, 57–67.
50. Noble, G. (1931) *The Biology of the Amphibia*. McGraw-Hill, New York.
51. Edwards, B. and Ruben, L. (1982) Aspects of amphibian immunity. In *Animal Models of Immunologic Processes.* Ha, J., Ed. Academic Press, London. pp. 255–286.
52. Rollins-Smith, L. and Cohen, N. (1996) Metamorphosis: an immunologically unique period in the life of the frog. In *Metamorphosis.* Gilbert, L., Atkinson, B., and Tata, J., Eds. Academic Press, San Diego. pp. 625–646.
53. Du Pasquier, L., Schwager, J., and Flajnik, M.F. (1989) The immune-system of Xenopus. *Ann. Rev. Immunol.* **7**, 251–275.
54. Izutsu, Y. and Yoshizato, K. (1993) Metamorphosis-dependent recognition of larval skin as non-self by inbred adult frogs (Xenopus-Laevis). *J. Exp. Zool.* **266**, 163–167.
55. Izutsu, Y., Tochinai, S., Maeno, M., Iwabuchi, K., and Onoe, K. (2002) Larval antigen molecules recognized by adult immune cells of inbred *Xenopus laevis*: partial characterization and implication in metamorphosis. *Dev. Growth Differ.* **44**, 477–488.
56. Ruben, L., Clothier, R., Horton, J., and Balls, M. (1989) Amphibian metamorphosis: An immunologic opportunity!, *Bioessays.* **10**, 8-12.
57. Rollins-Smith, L. (1998) Metamorphosis and the amphibian immune system. *Immunol. Rev.* **166**, 221–230.
58. Ono, M. and Tochinai, S. (1995) Demonstration of cells possessing tolerance-inducing activity in *Xenopus laevis* rendered tolerant perimetamorphically. *Transplantation* **60**, 66–70.
59. Mak, T. and Saunders, M. (2006) *The Immune Response - Basic and Clinical Principles.* Elsevier, Amsterdam.
60. Mescher, A.L. and Neff, A.W. (2005) Regenerative capacity and the developing immune system. In *Regenerative Medicine I: Theories, Models and Methods.* Yannas, I.V., Ed. Springer. pp. 39-66.
61. Sicard, R. and Lombard, M. (1989) Epimorphic regeneration and the immune system. In *Recent Trends in Regeneration Research.* Kiortsis, V., Koussoulakis, S., and Wallace, H., Eds. Plenum, New York. pp. 107–119.
62. Castell-Rodriguez, A.E., Hernandez-Penaloza, A., Sampedro-Carrillo, E.A., Herrera-Enriquez, M.A., Alvarez-Perez, S.J., and Rondan-Zarate, A. (1999) ATPase and MHC class II molecules co-expression in Rana pipiens dendritic cells. *Dev. Comp. Immunol.* **23,** 473–485.
63. Du Pasquier, L. and Flajnik, M.F. (1990) Expression of MHC class II antigens during *Xenopus* development. *Dev. Immunol.* **1**, 85–95.
64. Kaplan, D., Jenison, M., Saeland, S., Shlomchik, W., and Shlomchik, M. (2005) Epidermal Langerhans cell-deficient mice develop enhanced contact hypersensitivity. *Immunity* **23**, 611–620.
65. Del Rio-Tsonis, K., Tsonis, P.A., Zarkadis, I.K., Tsagas, A.G., and Lambris, J.D. (1998) Expression of the third component of complement, C3, in regenerating limb blastema cells of urodeles. *J. Immunol.* **161,** 6819–6824.
66. Kimura, Y., Madhavan, M., Call, M.K., Santiago, W., Tsonis, P.A., Lambris, J.A., and Del Rio-Tsonis, K. (2003) Expression of complement 3 and complement 5 in newt limb and lens regeneration. *J. Immunol.* **170**, 2331–2339.
67. Morais da Silva, S., Gates, P.B., and Brockes, J.P. (2002) The newt ortholog of CD59 is implicated in proximodistal identity during amphibian limb regeneration. *Dev. Cell* **3**, 547–555.
68. Nauta, A.J., Roos, A., and Daha, M.R. (2004) A regulatory role for complement in innate immunity and autoimmunity. *Int. Arch. Allergy Immunol.* **134**, 310–323.
69. Lee, J.Y., Atochina, O., King, B., Taylor, L., Elloso, M., Scott, P., and Rossman, M.D. (2000) Beryllium, an adjuvant that promotes gamma interferon production. *Infect. Immun.* **68**, 4032–4039.
70. Tsonis, P. (1996) *Limb Regeneration*. Cambridge University Press, Cambridge.

71. Shepherd, A.J., Downing, J.E.G., and Miyan, J.A. (2005) Without nerves, immunology remains incomplete - in vivo veritas. *Immunology* **116**, 145–163.
72. Paus, R., Theoharides, T.C., and Arck, P.C. (2006) Neuroimmunoendocrine circuitry of the 'brain-skin connection'. *Trends Immunol.* **27**, 32–39.
73. Harty, M., Neff, A.W., King, M.W., and Mescher, A.L. (2003) Regeneration or scarring: an immunologic perspective. *Dev. Dynam.* **226**, 268–279.

## BIOSKETCHES

Drs. Mescher and Neff are both Professors of Anatomy and Cell Biology in the Indiana University School of Medicine and members of the Indiana University Center for Regenerative Biology and Medicine. Each has 30 years' experience working on various aspects of amphibian embryogenesis or limb regeneration. They are currently collaborating with others in the Center on a global analysis of gene expression during *Xenopus* limb regeneration using microarray technology.
